# No Increased Risk of Herpes Zoster Found in Cirrhotic Patients: A Nationwide Population-Based Study in Taiwan

**DOI:** 10.1371/journal.pone.0093443

**Published:** 2014-04-03

**Authors:** Ping-Hsun Wu, Yi-Ting Lin, Chun-Nan Kuo, Wei-Chiao Chang, Wei-Pin Chang

**Affiliations:** 1 Division of Nephrology, Department of Internal Medicine, Kaohsiung Medical University Hospital, Kaohsiung, Taiwan; 2 Department of Family Medicine, Kaohsiung Medical University Hospital, Kaohsiung, Taiwan; 3 Graduate Institute of Medicine, College of Medicine, Kaohsiung Medical University, Kaohsiung, Taiwan; 4 Department of Pharmacy, Taipei Medical University-Wanfang Hospital, Taipei, Taiwan; 5 Department of Clinical Pharmacy, School of Pharmacy, Taipei Medical University, Taipei, Taiwan; 6 Department of Healthcare Management, Yuanpei University, Hsinchu, Taiwan; Hannover Medical School, Germany

## Abstract

**Background:**

The association between liver cirrhosis (LC) and herpes zoster has rarely been studied. We investigated the hypothesis that LC, known as an immunodeficiency disease, may increase the risk of herpes zoster using a national health insurance database in Taiwan.

**Materials and Methods:**

The study cohort included cirrhotic patients between 1998 and 2005 (n* = *4667), and a ratio of 1∶5 randomly sampled age- and gender-matched control patients (n* = *23,335). All subjects were followed up for 5 years from the date of cohort entry to identify whether or not they had developed herpes zoster. Cox proportional-hazard regressions were performed to evaluate 5-year herpes zoster-free survival rates.

**Results:**

Of all patients, 523 patients developed herpes zoster during the 5-year follow-up period, among whom 82 were LC patients and 441 were in the comparison cohort. The adjusted hazard ratio (AHR) of herpes zoster in patients with LC was not higher (AHR: 0.77, 95% confidence interval: 0.59–1.01, *p* = 0.06) than that of the controls during the 5-year follow-up. No increased risk of herpes zoster was found in LC patients after stratification by age, gender, urbanization level, income, geographic region, and all comorbidities.

**Conclusions:**

This large nationwide population-based cohort study suggests that there is no increased risk for herpes zoster among people who have LC compared to a matching population.

## Introduction

Herpes zoster is caused by spontaneous reactivation of a latent varicella-zoster virus (VZV) that resides in sensory ganglia and dorsal nerve roots following a varicella infection [1]. The disease usually manifests as painful vesicular skin lesions, which are limited to 1 to 3 dermatomes, and related neurological disorders. Several neurologic complications may be found, including post-herpetic neuralgia, ventriculitis, vasculopathy, cranial nerve palsies, encephalitis, and myelitis [2]. The most common complication is post-herpetic neuralgia, which results in functional disability and a poor quality of life. In addition, epidemiological studies also demonstrated the risk of stroke or cancer increases among patients who suffer from herpes zoster [3]–[6]. Therefore, herpes zoster has great impacts on the health of adults and health systems. The incidence and severity increase after the age of 50 years [7]–[10]. The incidence of herpes zoster is 1.2–4.9 cases per 1000 person-years in the general population [10]–[12]. Old age [13], diabetes mellitus (DM) [14], chronic renal failure (CRF) [15], [16], and chronic obstructive pulmonary disease (COPD) [17] are established risk factors for herpes zoster. Herpes zoster risk also increases with immunocompromised diseases, such as human immunodeficiency virus (HIV) infection [18], [19], systemic lupus erythematous (SLE) [20], and rheumatoid arthritis (RA) [21]–[23], as well as transplant recipients [24]. Although the mechanism of reactivation of latent VZV remains unclear, decreasing cellular immunity to VZV predisposes one to the recurrence of herpes zoster [25], [26].

Patients who develop liver cirrhosis (LC) exhibit acquired immune dysfunction because of poor homeostasis and malnutrition. Most of the host defense systems are compromised in cirrhotic patients, including antigen-specific and nonspecific functions, clearance capacities of the reticuloendothelial system, and macrophage, neutrophil, and lymphocyte functions [27]. Monocyte spread, chemotaxis, bacterial phagocytosis, and bacterial killing significantly deteriorate in cirrhotic patients [28]. Bacterial and fungal infections are more common and virulent in patients with cirrhosis than in the overall population [28]. In addition, LC can also be considered a risk of viral infection, such as cytomegalovirus [29], [30]. It is reasonable to hypothesize that the immune dysregulation found in LC may put patients at higher risk of developing herpes zoster. However, data are limited regarding the risk of herpes zoster among patients with LC. The goal of our study was to investigate whether patients with LC have a higher incidence of herpes zoster than the general population. This study provides unique data based on the Longitudinal Health Insurance Database (LHID).

## Methods

### Database

National Health Insurance (NHI) is a mandatory health insurance program in Taiwan that provided comprehensive coverage for medical for care up to 99% of the population in 2009. Because NHI is a single-payer compulsory program that covers all forms of health care for residents in Taiwan, the NHIRD comprehensively includes claim data on both outpatient and inpatient services for nearly the entire 23.7 million population of this country. All claims data are collected in the NHI Research Database (NHIRD) and are managed by the Taiwan National Health Research Institutes (NHRI). Data files in the NHIRD include all ambulatory claims, inpatient claims, details of ambulatory care and inpatient orders, and prescriptions dispensed at contracted pharmacies. Data used to perform the analyses conducted in this study were retrieved from the LHID 2005 (LHID2005), a subset of the NHIRD. The LHID2005 consists of all the original medical claims for 1,000,000 enrollees' historical ambulatory data and inpatient care data under the Taiwan NHI program from 1997 to 2010, and the database was created and is publicly released to researchers. The NHRI reported that there were no statistically significant differences in age or gender between the randomly sampled group and all beneficiaries of the NHI program. To maintain claims data accuracy, the NHI’s routine practice of performing cross-checks and validations of medical claims ensures the accuracy of the NHIRD diagnostic coding. Because we used de-identified secondary data released to the public for research purposes, our study was exempt from full review by the Institutional Review Board after consultation with the Director of the Kaohsiung Medical University Institutional Review Board.

### Study Population

We used a study cohort and a comparison cohort to examine the relationship between LC and herpes zoster. We identified 4667 first-time hospitalizations with a discharge diagnosis of LC or patients who had at least have two ambulatory care visits for LC (International Classification of Disease, 9^th^ Revision, Clinical Modification (ICD-9-CM) codes 571.2, 571.5, 571.6) between January 1998 and December 2005. The date of the initial diagnosis of LC was assigned as the index date for each LC patient. To the improve data accuracy, the LC selection criteria required that all cases with the ICD-9 code be assigned by an internist. We also established selection criteria for herpes zoster patient. We only included herpes zoster cases in this study if they received ≥2 herpes zoster diagnoses for ambulatory care visit or ≥1 diagnose for inpatient care, and the ICD-9 code was assigned by a dermatologist.

Our study used a study cohort and comparison cohort to examine the relationship between LC and herpes zoster. Each LC cohort patient was matched based on age, gender, and index year to five randomly identified beneficiaries without LC to build the comparison cohort. Patients diagnosed with herpes zoster (ICD-9-CM codes 053–053.9) before index date were excluded from both cohorts. We also identified relevant comorbidities, including hypertension (ICD-9-CM codes 401–405), DM (ICD-9-CM codes 250), hyperlipidemia (ICD-9-CM codes 272), HIV (ICD-9-CM codes 042), hepatitis B (ICD-9-CM codes 070.2,070.3, V02.61), hepatitis C (ICD-9-CM codes 070.41, 070.44, 070.51, 070.54, V02.62), organ transplantation (ICD-9-CM codes 996, V042), chronic renal failure (ICD-9-CM codes 585), SLE (ICD-9-CM codes 710), RA (ICD-9-CM 714), COPD (ICD-9-CM 491, 492, 496), cancer (ICD-9-CM codes 140–208), and alcoholism-associated disorders (ICD-9-CM codes 291, 303, 305.0, 357.5, 425.5, 571.0, 571.1, 571.2, 571.3, 980.0, V11.3).

### Level of Urbanization

For the urbanization level in our study, all 365 townships in Taiwan were stratified into 4 levels according to standards established by the Taiwanese NHRI based on a cluster analysis of the 2000 Taiwan census data, with 1 referring to the most urbanized area and 4 referring to the least urbanized. The criteria on which these strata were determined included the population density (persons/km^2^), the number of physicians per 100,000 people, the percentage of people with a college education, the percentage of people over 65 years of age, and the percentage of agricultural workers.

### Statistical Analysis

All data processing and statistical analyses were performed with the Statistical Package for Social Science (SPSS) software, vers. 18.0 (SPSS, Chicago, IL, USA) and SAS vers. 8.2 (SAS System for Windows, SAS Institute, Cary, NC, USA). Pearson *X* ^2^ tests were used to compare differences in geographic location, monthly income, and urbanization level of patients’ residences between the study and comparison groups. We also performed a survival analysis using the Kaplan-Meier method, and used the log-rank test to compare survival distributions between cohorts. The survival period was calculated for patients who suffered from LC until an occurrence of hospitalization, an ambulatory visit for herpes zoster, or the end of the study period (December 31, 2010), whichever came first. After adjusting for urbanization level, monthly income, region, and comorbidities as potential confounders, we performed a Cox proportional-hazards analysis stratified by gender, age group, and index year to examine the risk of herpes zoster during the 5-year follow-up in both cohorts. We further classified the duration of follow up period in both groups. Hazard ratios (HRs) and 95% confidence intervals (CIs) were calculated to quantify the risk of herpes zoster. The results of comparisons with a two-sided *p* value of <0.05 were considered to represent statistically significant differences.

### Ethical Approval

Insurance reimbursement claims data used in this study were from Taiwan’s NHIRD, which is available for research purposes. This study was conducted in accordance with the *Helsinki Declaration*. This study was also evaluated and approved by the Kaohsiung Medical University’s Institutional Review Board (KMUH-IRB-EXEMPT-20130059).

## Results

**Figure 1:**
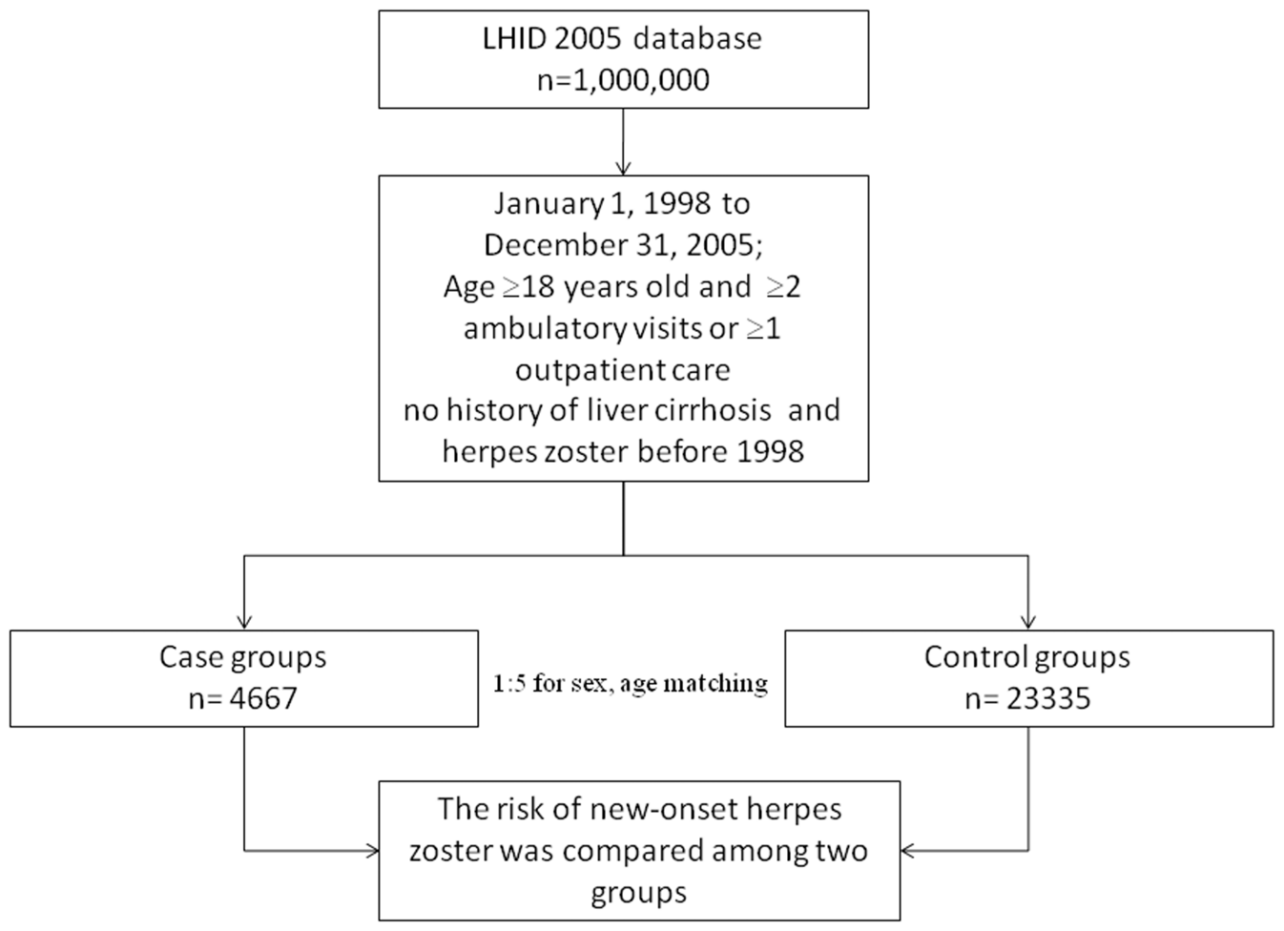
Flow chart of the selection of study subjects and control subjects from the National Health Insurance Research Database in Taiwan.

The research design of this study is shown in Figure 1. The LC cohort contained 4667 patients, and 23,335 patients were included in the comparison cohort. Distributions of demographic characteristics and comorbidities for the LC and comparison cohorts are shown in Table 1. Hypertension (*p*<0.001), DM (*p*<0.001), organ transplantation (*p*<0.001), hepatitis B (*p*<0.001), hepatitis C (*p*<0.001), CRF (*p*<0.001), SLE (*p*<0.001), RA (*p* = 0.004), COPD (*p*<0.001), cancer (*p*<0.001), and alcoholism (*p*<0.001) were more prevalent in the LC cohort than the comparison cohort. We also found that cases had a greater tendency to have a lower monthly income (*p*<0.001), to reside in central, southern and eastern Taiwan, and to reside in less-urbanized communities (*p*<0.001) compared to controls.

**Table 1:** Demographic characteristics of selected patients, stratified by the presence/absence of liver cirrhosis in 1998–2005 (n* = *28,002).

	Patients with liver cirrhosis(n = 4667)		Patients without liver cirrhosis(n = 23,335)		*p* value
	n	%	n	%	
**Gender**					1
Male	3236	69.3	16,180	69.3	
Female	1431	30.7	7155	30.7	
**Age (years)**					1
18–39	797	17.1	3985	17.1	
40–49	1046	22.4	5230	22.4	
50–59	1016	21.8	5080	21.8	
60–69	938	20.1	4690	20.1	
≥70	870	18.6	4350	18.6	
**Follow-up, year, mean (SD)**					0.22
	4.95	0.43	4.96	0.38	
**Urbanization level**					<0.001
1 (most urbanized)	1112	23.8	7483	32.1	
2	1326	28.4	6397	27.4	
3	765	16.4	3744	16.0	
4 (least urbanized)	1464	31.4	5711	24.5	
**Monthly income** *					<0.001
0	1063	22.8	4890	21.0	
NT$ 1–15,840	862	18.5	3405	14.6	
NT$ 15,841–25,000	2003	42.9	9515	40.8	
≧NT$ 25,001	739	15.8	5525	23.7	
**Geographic region**					<0.001
Northern	1787	38.3	10,942	46.9	
Central	1435	30.7	5802	24.9	
Southern	1124	24.1	5406	23.2	
Eastern	321	6.9	1185	5.1	
**Hypertension**					<0.001
Yes	2804	60.1	12,930	55.4	
No	1863	39.9	10,405	44.6	
**Hyperlipidemia**					0.67
Yes	2004	42.9	10,100	43.3	
No	2663	57.1	13,235	56.7	
**Diabetes**					<0.001
Yes	2168	46.5	7376	31.6	
No	2499	53.5	15,959	68.4	
**HIV**					0.02
Yes	9	0.2	27	0.1	
No	4658	99.8	23,308	99.9	
**Organ transplantation**					<0.001
Yes	239	5.1	642	2.8	
No	4428	94.9	22693	97.2	
**Hepatitis B**					<0.001
Yes	1865	40.0	2653	11.4	
No	2802	60.0	20,682	88.6	
**Hepatitis C**					<0.001
Yes	1375	29.5	1114	4.8	
No	3292	70.5	22,221	95.2	
**Chronic renal failure**					<0.001
Yes	636	13.6	1504	6.4	
No	4031	86.4	21,831	93.6	
**SLE**					0.01
Yes	35	0.7	109	0.5	
No	4632	99.3	23,226	99.5	
**Rheumatoid arthritis**					0.004
Yes	341	7.3	1439	6.2	
No	4326	92.7	21,896	93.8	
**COPD**					<0.001
Yes	2499	53.5	10,688	45.8	
No	2168	46.5	12,647	54.2	
**Cancer**					<0.001
Yes	2118	45.4	3227	13.8	
No	2549	54.6	20,108	86.2	
**Alcoholism**					<0.001
Yes	605	13.0	282	1.2	
No	4062	87.0	23,053	98.8	

Footnote: SD, standard deviation; HIV, human immunodeficiency virus; SLE, systemic lupus erythematous; COPD, chronic obstructive pulmonary disease.

*The average exchange rate in was US$1.00 ≈ New Taiwan (NT) $32.6.

**Figure 2:**
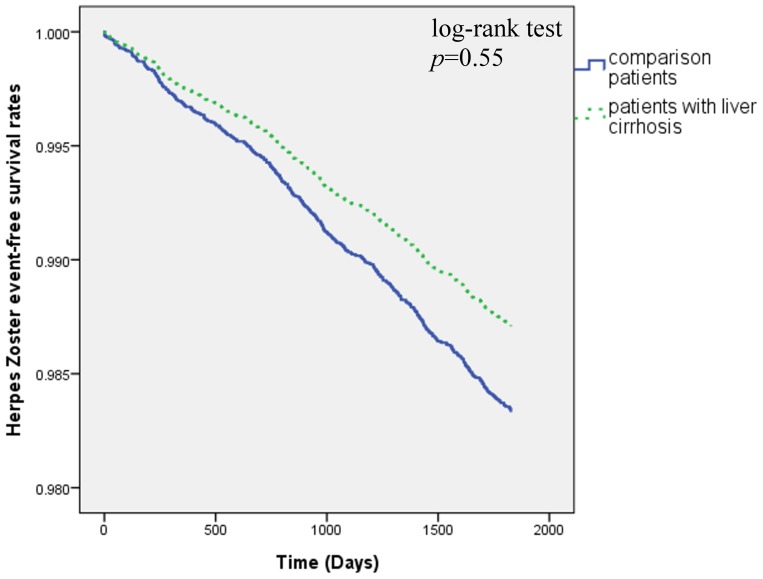
Herpes zoster-free survival rates for patient with liver cirrhosis and comparison groups in 1998 –**2005.**

There were 523 patients diagnosed with herpes zoster during the 5-year follow-up, including 82 LC patients (1.8%) and 441 patients in the comparison cohort (1.9%). The Kaplan-Meier survival curves are shown in Figure 2. The curves demonstrate no significant difference in herpes zoster-free survival rates between the LC and comparison cohorts (log-rank test *p = *0.55).

The Cox regression analysis showed that the crude HR for herpes zoster did not significantly differ between the LC and comparison cohorts (crude HR: 0.77, 95% CI: 0.59–1.01, *p* = 0.55). After adjusting for potential confounders, our results still showed no statistically difference in the risk of herpes zoster (adjusted HR (AHR): 0.77, 95% CI: 0.59–1.01, *p* = 0.06) between LC and non-LC patients (Table 2).

**Table 2:** Hazard ratios (HRs) and 95% confidence intervals (CIs) of herpes zoster among liver cirrhosis patients during the 5-year follow-up period from the index ambulatory visit or inpatient care in 1998–2005.

	Total		Patients with livercirrhosis		Patients withoutliver cirrhosis	
Development of herpeszoster	No.	(%)	No.	(%)	NO.	(%)
5-year follow-up period						
Yes	523	1.9	82	1.8	441	1.9
No	27,479	98.1	4585	98.2	22,894	98.1
Crude HR (95% CI)				0.93 (0.74–1.18)	1	
Adjusted HR (95% CI)				0.77 (0.59–1.01)	1	

Adjustments are made for patients’ gender, age, urbanization level, geographic region, monthly income, hypertension, diabetes, human immunodeficiency virus, organ transplantation, hepatitis B, hepatitis C, chronic renal failure, systemic lupus erythematous, rheumatoid arthritis, chronic obstructive pulmonary disease, cancer, and alcoholism.

**Figure 3:**
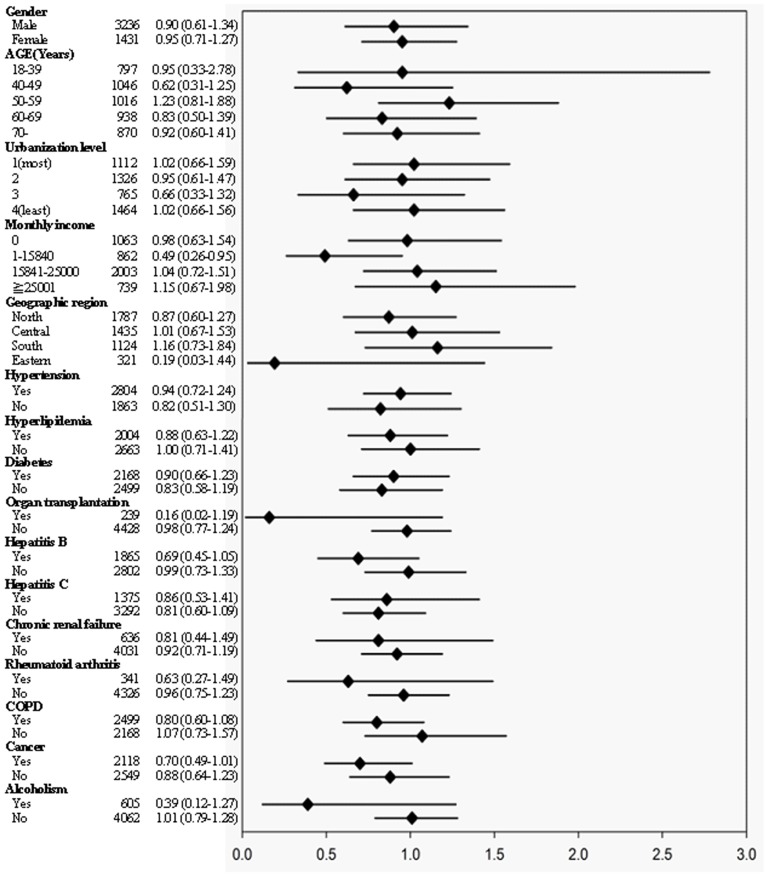
Multivariate stratified analyses of the association of liver cirrhosis with herpes zoster. In each stratum, liver cirrhosis was not statistically associated with herpes zoster.

We further investigated whether LC is a time-dependent risk factor for herpes zoster and divided LC patients into 3 groups according to the follow-up period. For the three different follow-up periods, there was still no statistical significance between the case and comparison groups. The 1 and 3-year follow-up period demonstrated no statistical significance (AHR: 1.16; 95% CI: 0.67–2.00, *p* = 0.60; AHR: 0.83; 95% CI: 0.59–1.17, *p* = 0.28) (Table 3). We also further conducted a multivariate stratified analysis, and results revealed that LC was not associated with a risk of herpes zoster in any subgroup (Figure 3).

**Table 3:** Hazard ratios (HRs) and 95% confidence intervals (CIs) of herpes zoster among liver cirrhosis patients during the 1-, 3-, and 5-year follow-up periods from the index ambulatory visit or inpatient care in 1998–2005.

	1-year follow-up period		3-year follow-up period		5-year follow-up period	
Development of herpes zoster	Patients with liver cirrhosis	Comparison cohort	Patients with liver cirrhosis	Comparison cohort	Patients with liver cirrhosis	Comparison cohort
Yes (%)	21 (0.4)	83 (0.4)	53 (1.1)	252 (1.1)	82 (1.8)	441 (1.9)
No (%)	4646 (99.6)	23252 (99.6)	4614 (98.9)	23,083 (98.9)	4585 (98.2)	22,894 (98.1)
Crude HR (95% CI)	1.27 (0.79–2.05)	1	1.05 (0.78–1.42)	1	0.93 (0.74–1.18)	1
Adjusted HR (95% CI)	1.16 (0.67–2.00)	1	0.83 (0.59–1.17)	1	0.77 (0.59–1.01)	1

Adjustments were made for patients’ gender, age, urbanization level, geographic region, monthly income, hypertension, diabetes, human immunodeficiency virus, organ transplantation, hepatitis B, hepatitis C, chronic renal failure, systemic lupus erythematous, rheumatoid arthritis, chronic obstructive pulmonary disease, cancer, and alcoholism.

## Discussion

Our study presents fundamental epidemiological data regarding herpes zoster among patients with LC. To our knowledge, this is the first cohort study to assess the link between LC and the risk of developing herpes zoster. While the validity of ICD-9-CM codes is known to vary widely, an ICD-9-CM diagnosis of herpes zoster and diagnosis by chart review in the Veterans Affairs health care system were in excellent agreement (κ = 0.92) [23]. We found that 1.8% of patients developed herpes zoster in the 5 years following an LC diagnosis. Our nationwide population-based study clearly demonstrated that after adjusting for potential confounders, patients with LC were not at an increased risk of herpes zoster. A further multivariate stratified analysis and different follow-up time analysis still showed no increased risk of herpes zoster among patients with LC.

Patients with suppressed cell-mediated immunity caused by immunosuppressive disorders or therapies, such as elderly people, patients with autoimmune disease, malignancy, or diabetes, are well known to have a higher risk of developing herpes zoster [13], [14], [21]–[23], [31]–[33]. Although the mechanism involved in reactivation of latent VZV remains unclear, there is evidence that a decline in cellular immunity to VZV predisposes one to the occurrence of herpes zoster [25], [26]. LC, which is related to lymphocyte and macrophage dysfunction and decreased production of proinflammatory cytokines, such as interferon-γ and tumor necrosis factor-α, may be linked to an increased virus infection risk [28]. The liver contains 90% of cells of the reticuloendothelial system (Kupffer cells and sinusoidal endothelial cells), which are essential for pathogen eradication. Monocyte spread, chemotaxis, and pathogen phagocytosis and killing significantly deteriorate in cirrhosis patients [34]. The phenomena of decreased neutrophil mobilization and phagocytic activity were correlated with the severity of liver disease [35]. Decreased phagocytic activity and the characteristic neutropenia, hyperammonemia, and hyponatremia are exacerbated by shortened neutrophil survival and a decreased opsonization capacity [36], [37]. LC is known as innate immunity dysfunction and increased infection risk by bacteria and fungi, and even the cytomegalovirus. In contrast, our cohort study did not show an association between LC and herpes zoster.

From post liver transplantation study, the herpes zoster risk was inconclusive [38], [39] and most believe immunosuppressive agents are more important than transplant *per se*. Besides, in Bajaj et al.’s study, most infection in liver cirrhosis patients were urinary tract infection, spontaneous bacterial peritonitis, spontaneous bacteremia and cellulitis [40]. Herpes zoster was not in the finding in the study. Our result showed the incidence in liver cirrhosis patient was not higher than control group. It was consisted with Bajaj et al.’s study. In McDonald et al.’s study, they found liver disease was not a risk factor for herpes zoster [23]. Although the immunity in liver cirrhosis patients is worse than healthy, other mechanism for virus reactivation may exist.

The present study has a number of strengths. First, this is a large-scale follow-up study using the well-established nationwide NHI research database. The study cohort is highly representative of the general Taiwanese population. The ascertainment of LC hospitalization and medical comorbidities is likely complete and accurate because the NHI is a compulsory and universal healthcare system. In addition, while racial differences are considered to be a factor that influences the risk of herpes zoster [13], approximately 98% of Taiwan’s residents are of Han Chinese ethnicity; this relatively homogenous population reduces potential confounding by race in our study. Furthermore, with the NHIRD, claims for each insured can be tracked across time. In the present study, all claims of different medical institutes during the study period were obtained for analysis. This avoided the bias of patients dropping out that occurs in most longitudinal studies and minimized the possibility of recall bias.

Nevertheless, several limitations should be recognized and discussed. First, because almost all herpes zoster and LC cases were diagnosed clinically without serologic confirmation or standardized procedures, diagnoses based on ICD-9-CM codes may be less accurate, and we could not discern the severity of LC based on the Child-Pugh classification score according to ICD-9 coding. However, the NHI Bureau in Taiwan randomly interviews patients and reviews medical records every year to confirm the validity of the diagnoses and quality of care by randomly sampling a certain percentage of claims from every hospital. It is generally believed that the NHIRD has acceptable quality and accuracy of disease coding for epidemiological analyses. Second, some patients with herpes zoster might have been missed in our database if they did not seek medical help, particularly if their symptoms were mild. Miscoding and misclassification could occur as potential biases. However, utilization of medical services in Taiwan is generally high because there are very low financial barriers to medical care with a very low copayment system. Patients are responsible for a copayment of only US$3∼15 each visit, so most patients with herpes zoster would seek medical attention after disease onset. Thus, the number of herpes zoster cases not included in NHIRD is likely to be small [3]. Third, the claims database lacks information on cigarette smoking, alcohol consumption, dietary habits, physical activities, environmental exposure, nutritional status, and family history, which may confound our findings. Fourth, LC is a highly lethal disease in the advanced stage. Therefore, death occurring prior to herpes zoster being diagnosed was considered a competing risk event. However, the death condition cannot be known from NHIRD, which may have biased our results. Fifth, the patients who had herpes zoster after the study period cannot be known from the database. It may be a bias for our result.

In summary, our study provides fundamental epidemiological data that the incidence of herpes zoster is not higher in LC patient in a nationally representative cohort followed-up for 5 years. Studies to find the mechanism for virus reactivation beyond immune insufficiency might be necessary.
